# Occurrence, Source Apportionment, and Ecological Risk of Typical Pharmaceuticals in Surface Waters of Beijing, China

**DOI:** 10.3390/toxics12030171

**Published:** 2024-02-23

**Authors:** Yonghao Huangfu, Qingshan Li, Weiwei Yang, Qingwei Bu, Lei Yang, Jianfeng Tang, Jie Gan

**Affiliations:** 1 School of Chemical & Environmental Engineering, China University of Mining & Technology-Beijing, Beijing 100083, China; 2 State Key Laboratory of Urban and Regional Ecology, Research Center for Eco-Environmental Science, Chinese Academy of Sciences, Beijing 100085, China; 3 Key Laboratory of Urban Environment and Health, Institute of Urban Environment, Chinese Academy of Sciences, Xiamen 361021, China; 4 Hunan Ecological and Environmental Monitoring Center, Changsha 410014, China

**Keywords:** pharmaceutical, the North Canal Basin, positive matrix factorization, source, ecological risk

## Abstract

Various studies have shown that the heavy use of pharmaceuticals poses serious ecological risks, especially in metropolitan areas with intensive human activities. In this study, the spatial distribution, sources, and ecological risks of 29 pharmaceuticals in 82 surface waters collected from the North Canal Basin in Beijing were studied. The results showed that the pharmaceutical concentrations ranged from not detected to 193 ng/L, with ampicillin being undetected while ofloxacin had a 100% detection frequency, which indicates the widespread occurrence of pharmaceutical pollution in the North Canal Basin. In comparison with other freshwater study areas, concentrations of pharmaceuticals in the North Canal Basin were generally at moderate levels. It was found that pharmaceutical concentrations were always higher in rivers that directly received wastewater effluents. Source analysis was conducted using the positive matrix factorization model. Combining the spatial pollution patterns of pharmaceuticals, it has been found that wastewater effluents contributed the most to the loads of pharmaceuticals in the studied basin, while in suburban areas, a possible contribution of untreated wastewater was demonstrated. Risk assessment indicated that approximately 55% of the pharmaceuticals posed low-to-high ecological risks, and combining the results of risk analyses, it is advised that controlling WWTP effluent is probably the most cost-effective measure in treating pharmaceutical pollution.

## 1. Introduction

Since the 20th century, with the rise in manufacturing levels and the implementation of healthcare policies, pharmaceuticals have been widely used to treat human diseases and promote livestock growth [1]. China is a major producer and consumer of pharmaceuticals, with per capita antibiotic use 5.5 and 7.7 times that of the United States and the United Kingdom, respectively [2]. Therefore, it has been observed that pharmaceutical concentrations in surface water are spread over a wide range (from ng/L to μg/L [3].

Even low pharmaceutical concentrations may have adverse effects on aquatic organisms. On the one hand, with the pseudo-persistence, pharmaceuticals in the natural environment can be ingested by non-target organisms, such as zooplankton or fish, and bioaccumulate through the food chain and food webs, which, in turn, can pose a risk to humans [4]. On the other hand, this can lead to the development of antibiotic-resistant bacteria, reduce pharmaceutical effectiveness, and pose a threat to aquatic life and even human health [5].

Humans gradually recognized the seriousness of pharmaceutical contamination and bound the norms of pharmaceutical use by designating regulations, e.g., the new veterinary regulation (Regulation (EU) 2019/6). However, there is still secondary contamination caused by the widespread use of reclaimed water containing pharmaceuticals [6]. Although control at source is a common measure of managing various pollutants, large amounts of manpower and material resources are required in order to achieve comprehensive management of pharmaceutical pollution due to the diversity of their sources [7,8]. Therefore, it is necessary to identify the major pollution sources, which can also help predict and treat pollution, especially in large metropolitan areas with complex sources of discharges, which are much more cost-effective.

To our knowledge, in recent years, exploring the occurrence of pharmaceuticals in drinking water and wastewater treatment plant (WWTP) effluents has become a hot research topic in the field of surface water environments, while less focus has been given to receiving streams, which are most heavily polluted by humans [9,10]. Therefore, investigations of the occurrence, distribution, sources, and risks of pharmaceuticals in the natural surface water environment are urgently required.

In this study, 88 surface water samples were collected in the North Canal Basin within Beijing based on collection activities in November 2021. The specific work is as follows: (1) investigate the characteristics of pharmaceutical pollution in the study basin; (2) quantify the source of the pharmaceuticals and the contribution of each source; (3) assess the ecological risks of pharmaceuticals in the study; and (4) combined with the pollution sources and ecological risk analyses, make recommendations for the prevention the pharmaceutical pollution.

## 2. Materials and Methods

### 2.1. Pharmaceutical and Reagents

Table S1 provides detailed information for 29 target pharmaceuticals and 5 internal standards. The target pharmaceuticals were categorized as follows: 7 sulfonamides (SAs), 3 tetracyclines (TCs), 5 fluoroquinolones (FQs), 4 macrolides (MLs), and 10 mixed categories of multiple pharmaceuticals (OTs). These pharmaceuticals were selected based on their dosage, environmental detection rates, and ecological risks [11]. Internal standards: roxithromycin-d7, chloramphenicol-d5, sulfamethazine-d4, carbamazepine-d10, norfloxacin-d5, and demeclocycline. Pharmaceutical standards and internal standards were obtained from J&K Scientific Ltd. (Beijing, China). Acetonitrile and methanol (LC-MS grade) were obtained from Fisher Chemical, Inc. (Waltham, MA, USA). Formic acid and ammonium acetate were purchased from Sinopharm Chemical Reagent Co. (Shanghai, China). Analyte stock solutions are stored at −20 °C.

### 2.2. Sample Collection and Pretreatment

**Figure 1:**
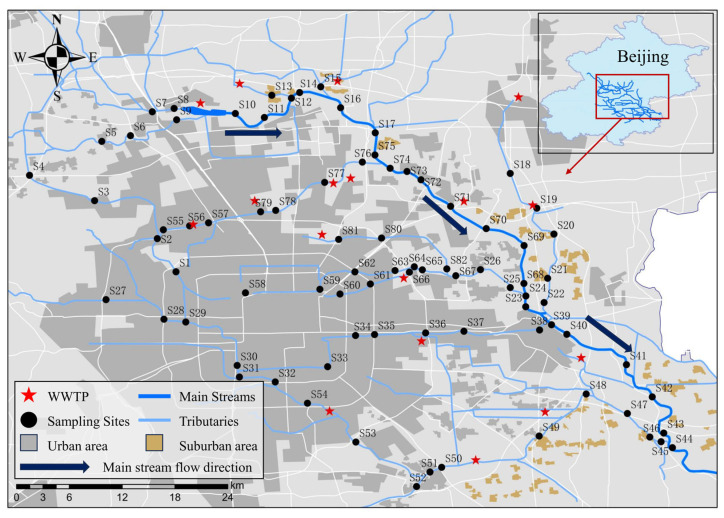
Sampling map of the study area.

A total of 82 water samples were collected from the North Canal Basin within the Beijing region in November 2021. The spatial distribution of the sampling sites is shown in Figure 1. At each sampling site, surface water samples were collected using 6 L polyethylene plastic buckets. Prior to sampling, the buckets were rinsed in the laboratory with tap water, deionized water, and methanol, respectively, and then moistened with river water three times. All water samples were collected approximately 20 cm below the water surface near the riverbank. The plastic buckets filled with water were sealed with cap and sealing film and wrapped in aluminum foil to shield them from light. Subsequently, they were stored at 4 °C, transported to the laboratory, and processed within 24 h. More details about the study sites and sample pretreatment are provided in the Supporting Information.

### 2.3. Instrumental Analysis

Samples were analyzed using a high-performance liquid chromatograph–triple quadrupole mass spectrometer coupled system (LCMS-8040, Shimadzu, Japan). Shim-pack XR-ODS columns were used to separate the target pharmaceuticals. Ultrapure water containing 0.2% formic acid (*v*/*v*) was used as mobile phase A, and acetonitrile was used as mobile phase B. The sample preparation method and instrumental analysis conditions were referenced from our previous work [12]. Detailed information is presented in Table S1.

### 2.4. Quality Assurance and Quality Control (QA/QC)

In this study, five internal standards were utilized for quantifying the target pharmaceuticals, including roxithromycin-d7, chloramphenicol-d5, sulfamethazine-d4, carbamazepine-d10, and demeclocycline. Parallel samples were employed during testing to assess experimental stability, with the relative deviation of parallel samples required to be below 30%. To ensure experimental accuracy and select target pharmaceuticals with minimal matrix effects, blank spiking and matrix spiking recovery experiments were conducted. Deionized water and water samples from randomly selected sites (every 10 sampling sites) were utilized for blank spiking and matrix spiking experiments, respectively. Recovery rates for the 29 pharmaceuticals ranged from 34.5% to 184%, with standard deviations ranging from 0.72% to 31.2% for the same pharmaceutical. Further details, such as standard curves and method detection limits (MDL) for each pharmaceutical, are presented in Table S3, along with the treatment of non-detected pharmaceuticals in ecological risk calculations using 1/2 MDL.

### 2.5. Positive Matrix Factorization (PMF) Model

PMF is a multivariate analysis tool used to identify pollution sources based on the detected concentrations of input pollutants. The PMF model was initially developed in 1994 by Paatero and Tapper and was originally used to identify the sources of trace metals in water [13].

There are two important parameters in the PMF model. One of them is the objective function Q, which is defined to minimize in the model. The model iterates multiple times to select the best solution, and the best solution is determined by the minimum value of Q. The other one is the uncertainty *u_ij_*, whose calculation is not fixed.

The functional equation for the uncertainty *u_ij_* is given below [14]:

(1) $$u_{ij}=\left\{\begin{array}{c} \frac{5}{6}\times MDL,x_{ij}\leq MDL\\ \sqrt{{\left(\sigma_{i}\times x_{ij}\right)}^{2}+{\left(MDL\right)}^{2}},x_{ij}>MDL, \end{array}\right.$$

where *σ_i_* is the relative standard deviation of the concentration of the pharmaceutical *i*, and *x_ij_* means the concentration matrix of the *j*th pharmaceuticals in the *i*th sample.

In this study, EPA PMF V5.0 software was utilized. Before initiating the PMF model simulation, it is essential to conduct a statistical analysis of the detected data [15]. Weights are assigned to the pharmaceuticals based on their uncertainties, with three levels— “strong”, “weak”, and “bad”—after the concentration and uncertainty data are input into the PMF model. To obtain the best simulation results, the model is run 200 times, which is more than the minimum requirement (20 times). The sources of pharmaceuticals simulated can range from 2 to 8 pathways, and the number of sources corresponding to the best Q value determines the number of pharmaceutical sources.

### 2.6. Ecological Risk Assessment

The risk quotient (*RQ*) method was utilized to assess the potential harm to the ecosystem caused by pharmaceuticals through ecological risk assessment [16]. The calculation method for *RQ* is as follows:

(2) $${RQ}_{i}=\frac{{MEC}_{i}}{{PNEC}_{i}},$$

where *RQ_i_* is the ecological risk quotient for pharmaceutical *i*, *MEC_i_* is the detected concentration of pharmaceutical *i* in the water, and *PNEC_i_* is the predicted no-effect concentration of pharmaceutical *i* in aquatic organisms.

In the present study, the species sensitivity distribution (SSD) method was used to derive the corresponding *PNEC* values when there was sufficient toxicity data for the pharmaceuticals, and the assessment factor (AF) method was used for the rest [17,18]. The *PNEC* values were predicted by ECOSAR, a computerized structure–activity relationship for aquatic toxicity, when the toxicity was not available. The derivation of the SSD method follows the technical guidelines published by the Ministry of Ecology and Environment of the People’s Republic of China and is calculated with recommendation software EEC-SSD (version 1.0) [17]. To avoid the phenomenon of overprotection, chronic ecotoxicity, which can protect 95% of organisms from being affected, was utilized to calculate the *PNEC* value. The PNEC value derived using the AF method is calculated by dividing the median effective concentration (*EC*_50_) or no observed effect concentration (*NOEC*) value of the most sensitive biological endpoint for pharmaceuticals by a deterministic value selected based on technical guidance from the European Commission using the following equation:

(3) $${PNEC}_{i}=\frac{{EC}_{50-i}}{{AF}_{i}}\;or\;{PNEC}_{i}=\frac{{NOEC}_{i}}{{AF}_{i}},$$

where *EC*_50−*i*_ is the median effective concentration for pharmaceutical *i*, *NOEC_i_* is the *NOEC* value of the most sensitive biological endpoint for pharmaceutical *i*, and *AF_i_* is the assessment factor for pharmaceutical *i*. The *AF* values are selected based on the criteria provided in Table S3. To better elucidate the risks of pharmaceuticals, the *RQ* is categorized into four levels: *RQ* < 0.01: insignificant risk; 0.01 ≤ *RQ* < 0.1: low risk; 0.1 ≤ *RQ* < 1: moderate risk; *RQ* ≥ 1: high risk [19].

## 3. Results and Discussion

### 3.1. Occurrence of Selected Pharmaceuticals

Among the 29 pharmaceuticals, 28 were detected in the North Canal Basin (Table 1), with concentrations ranging from N.D. to 193 ng/L, except for AMP, which was not detected. OFX had a 100% detection frequency, which indicated the widespread occurrence of pharmaceutical pollution in the North Canal Basin.

Among the five drug classes, SAs contributed 19% of the total concentration, with a mean concentration and detection frequency of 5.77 ng/L and 65%, respectively. TMP, which is a synergist of SAs, and dominates SAs. TCs had the lowest mean detection frequency of 37%, with a mean concentration of 4.60 ng/L, and contributed only 9% of the total concentration. In the TCs, TC played a critical role, having the uppermost mean concentration and detection frequency, respectively. In addition, FQs contributed 29% of the total drug concentration, with the mean concentration and mean detection frequency of 12.4 ng/L and 52%, respectively. As a widely used human and veterinary medicine, NFX and OFX were found to have the highest concentration and the highest detection frequency, respectively, and the annual use of both in China exceeds 5 × 10^6^ kg [2]. MLs had the highest mean detection frequency and mean concentration of 88% and 12.4 ng/L, respectively, accounting for 29% of the total pharmaceutical concentration. CLR was the predominant component of the MLs category, with the highest mean concentration and detection frequency in the MLs, respectively. OTs had the lowest mean concentration of 4.36 ng/L but contributed 22% of the total antibiotic concentration, which may be the result of the large number of pharmaceutical classes (10) included in this classification. Among OTs, DF dominated in the North Canal Basin, with the uppermost mean concentration and detection frequency. In summary, TMP, TC, NFX, OFX, CLR, and DF were identified as the predominant substances among respective pharmaceutical categories due to their remarkable mean concentration and detection frequency.

Concentrations of pharmaceuticals in the North Canal Basin were generally at a moderate level compared to the six remarkable pharmaceutical concentrations in other freshwater study areas (Table S5). The mean concentration of TMP was higher than those found in the Nanming River, Chao Lake, and three lakes of Sweden, while lower than those in Xiaoqing River. The mean concentration of TC is significantly lower than that reported in Chao Lake, but much higher than that found in the Nanming River and Xiaoqing River. The mean concentration of OFX varies significantly in different study areas, which was the third highest in the study, lower than in the Nanming River and Xiaoqing River, but higher than Chao Lake. The pollution by NFX was the most serious, and the mean concentration was significantly higher than the Liao River and Yangtze River and similar to that detected in the Nanming River. For CLR, the mean concentration was the second highest, slightly lower than the Xiaoqing River, and significantly higher than the Yangtze River, Sangong River, and the lakes of Sweden. The mean concentration of DF was quite low when compared with Arkavathi Basin (India), Paraopeba River (Brazil), Zhangxi River, and Lu River, while slightly higher than the three lakes of Sweden. In addition to these remarkable pharmaceuticals, mean concentrations of SPD, SMM, SMZ, SMZ-2, FFC, and GEM were higher than those detected in other study areas, while PEF and AMP were lower in the North Canal Basin. Furthermore, the mean concentration of other pharmaceuticals was similar to the detected level of other study areas.

### 3.2. Spatial Distribution of Pharmaceutical

**Figure 2:**
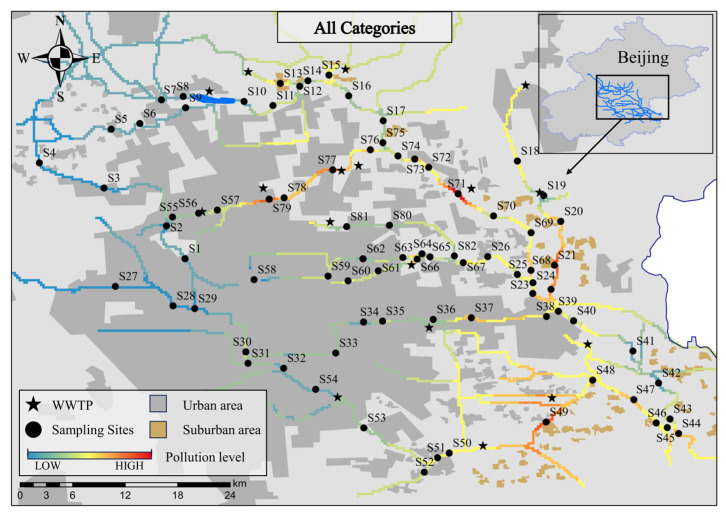
Interpolation map of inverse distance weights of all categories of pharmaceutical concentrations in the North Canal Basin.

Different categories of pharmaceuticals showed significant spatial differences in the North Canal Basin. As Figure 2 shows, more severe pharmaceutical contamination was found in receiving rivers, and higher total pharmaceutical concentrations for all categories were more widely detected in tributaries than in the main stream. The highest total pharmaceutical concentration (531.8 ng/L) was detected at sampling site S71, where a WWTP effluent was located 50 m upstream. Likewise, WWTP effluents are located a short distance upstream of sampling sites S77 and S79. Similarly, sampling sites S21 and S49 were located downstream of the catchment of a village discharge stream.

Among the five categories of pharmaceuticals, the concentrations in the tributaries and receiving rivers were higher than those detected in the main stream and non-receiving rivers (Figure S1). In the receiving rivers, the mean concentration of TCs in the tributaries differed significantly from that in the main streams, with the highest mean concentration of 340 ng/L in the Lin Gou River being 17.3 times higher than that in the North Canal. Differences between tributaries and main streams for other categories of pharmaceuticals were not significant, with tributaries having the highest concentrations of SAs, FQs, MLs, and Ots, being 2.70, 2.40, 3.30, and 4.59 times higher than the main streams, respectively. Furthermore, SAs, TCs, and OTs had more severe pollution occurring in suburban areas, while FQs and MLs were in urban areas. This could be due to FQs and MLs being used to treat human disease, while FF of OTs, which made the concentration of OTs in suburban areas significantly higher than that in urban areas, similar to SAs and TCs, were widely used in veterinary medicine and pesticides.

Overall, in the North Canal Basin, the extensive use of pharmaceuticals and complex human activities in Beijing has led to non-negligible pharmaceutical pollution in rivers, especially the higher concentrations of pharmaceuticals detected in the receiving rivers, which pass through suburban areas and WWTP discharge sites. The former may be related to direct wastewater discharge, while the latter may be associated with WWTPs being the primary collection sites for human sewage in highly urbanized areas [20,21]. Furthermore, the elevated total pharmaceutical concentrations in the main stream may also be caused by the confluence of tributaries with high pharmaceutical concentrations.

### 3.3. Quantitative Source Apportionment of Pharmaceuticals

The concentration data were pre-analyzed using PMF, in which 10 pharmaceuticals were set as “bad”, and 82 data of 18 pharmaceuticals were analyzed at last. The data meet the data requirements of the PMF model, ensuring that the simulation results are credible. The number of runs was set to 200 to ensure the realism of the model simulation results. The best simulation result is obtained when the factor is set to 3 to obtain the minimum Q value. In the results, the R^2^ values for “Obs/Pred Scatter Plot” for most pharmaceuticals exceeded 0.5, with some values exceeding 0.72. This indicates that the predicted concentrations closely match the actual values in the “Base Model Runs”, demonstrating the reliability of the modeling results.

**Figure 3:**
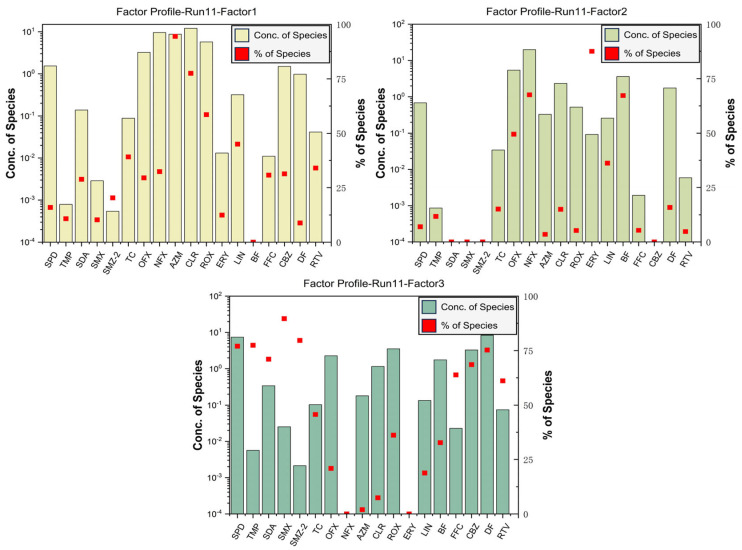
Contributions from each factor to pharmaceuticals.

According to the PMF model simulation results (Figure 3 and Figure S2), Factor 1 contributed to 40.9% of the pharmaceutical concentration, primarily driven by four pharmaceuticals: AZM (94.5%), CLR (77.5%), ROX (58.6%), and LIN (45.0%). In recent decades, due to the broad-spectrum antibacterial properties of LIN and MLs such as AZM, CLR, and ROX, which are effective against most Gram-positive bacteria, some Gram-negative bacteria, and specific atypical pathogens (such as mycoplasmas and chlamydiae) [22,23]. Unabsorbed pharmaceuticals are excreted into the natural environment through feces and urine. In previous investigations, the frequency and concentration of MLs detected in human urine could reach 74% and 10^5^ μg/L [24]. As the dominating domestic sewage catchment area in the urban, the detected concentration of four pharmaceuticals in the WWTP of Beijing from 10^2^ ng/L to several μg/L [25]. In addition, the centralized treatment efficiency of WWTPs was limited for CLR, ROX, and LIN, which means removal rates just from 76.3% to 84.4% in Beijing [26]. As per the previous research, current municipal wastewater treatment technologies are ineffective in removing LIN, with effluent concentrations in some cases exceeding influent concentrations [27,28]. Combined with the pharmaceutical concentration spatial distribution of factor 1, more serious pollution occurred near the WWTP (Figure S3). Therefore, factor 1 likely represents water discharged after WWTP treatment, i.e., WWTP effluent.

Factor 2 contributed 32.5% of the pharmaceutical concentration. OFX (49.6%), NFX (67.6%), ERY (87.6%), and BF (67.3%) exhibited higher loads in Factor 2. BF, one of the most commonly prescribed human lipid-lowering medications found in natural aquatic environments, shows a high detection rate in hospital discharge of medical wastewater and WWTP [26,29]. ERY and NFX are widely used as pharmaceuticals for treating inflammation and various bacterial infections [2]. High concentrations of ERY in medical wastewater were detected in Beijing, which was imported into WWTP at last [30]. Furthermore, OFX, as a broad-spectrum antibiotic, is widely used to reduce the incidence of disease and promote growth, which has a high detection frequency and concentration in livestock [31,32]. Combined with the pharmaceutical concentration spatial distribution of factor 2, the pollution levels are higher both near WWTP and suburban areas (Figure S3). As most categories of pharmaceuticals in factor 2 are used in clinical practice, it can be inferred that factor 2 represents uncertain sources and complex sources of mainly medical wastewater.

Factor 3 contributed 26.61% of the pharmaceutical concentration and mainly drove the concentrations of SPD (77.0%), TMP (77.4%), SDA (71.1%), SMX (89.7%), SMZ-2 (79.7%), TC (45.7%), FFC (63.8%), CBZ (68.6%), DF (75.3%), and RTV (61.1%). SAs have a wide antibacterial spectrum and broad applications in aquaculture, livestock, and medical fields, which were detected in high concentrations in suburban areas, especially near the fishing gardens and a WWTP of collecting village sewage, in this study [33,34,35,36]. TC is widely used for treating both human and animal diseases due to its broad-spectrum activity and cost-effectiveness, with a significant removal rate in WWTP of Beijing [30]. However, the highest concentration of TC was detected at S57, located downstream of an urban WWTP, although TC is more widely distributed in suburban areas. FFC is a broad-spectrum antibacterial agent primarily used in veterinary medicine to treat animal respiratory diseases; a significant concentration of FFC was detected near a village with an equestrian club [37]. CBZ and DF are extensively used in treating human diseases and are common residual pharmaceuticals in water bodies [38,39]. In addition, nearly 50% of CBZ and 40% of DF is metabolized out of the body into domestic sewage through urine [40,41]. In this study, compared with urban areas, the distribution of the two compounds was more extensive and the detected concentrations were higher in suburban areas. RTV is a highly effective pharmaceutical used to treat immune deficiency syndrome. Additionally, RTV has been widely used in the fight against Coronavirus Disease 2019 (COVID-19) [42]. The spatial distribution of RTV was similar to that of FFC, except significant concentrations were also detected at a small number of urban area sampling sites. The combined spatial distribution of pharmaceutical concentrations was analyzed, and Factor 3 caused more severe contamination, mainly in suburban areas (Figure S3). To sum up, Factor 3 is most likely to be a mixed source from the suburban area, which includes WWTP, aquaculture (fishing gardens), domestic sewage from villages, etc., located in the study area.

Among these three factors, the WWTP effluent was the dominant source of pharmaceutical pollution in the study area. As the largest artificial wastewater catchment area in the urban regions, WWTP receives sewage from different sources, and there is a large and diverse occurrence of pollutants in the WWTP effluent [22]. The total pharmaceutical concentration in uncertain sources is also significantly high, which probably mainly consists of medical wastewater. As the capital, Beijing has abundant medical and healthcare resources. According to statistics, there is a considerable influx of people from other regions seeking medical consultations and treatment in Beijing, with the number of patient visits to healthcare institutions in Beijing reaching 2.43 × 10^8^ in 2021 [43]. Furthermore, there are a lot of livestock in suburban areas, as well as pharmaceuticals unabsorbed or unused by patients, and livestock are discharged into the natural environment through complex pathways. Suburban area mixed sources accounted for the lowest proportion. As a highly urbanized city with a large population, Beijing has good coverage of municipal pipe networks. However, previous studies have shown that there may still be a portion of untreated domestic wastewater discharged into natural waters [44]. In addition, due to the limited number of sampling sites in this study, it is difficult to fully identify sources of pharmaceutical pollution in suburban areas, such as farmland drainage, which has been reported in other studies [45].

To sum up, the area of this study is only a part of Beijing, and the sources of pharmaceutical pollution in the city are complex. Among these, WWTP effluent contributes most of the pharmaceutical load, while in suburban areas, there may be pollution from untreated domestic wastewater.

### 3.4. Ecological Risk Assessment

**Figure 4:**
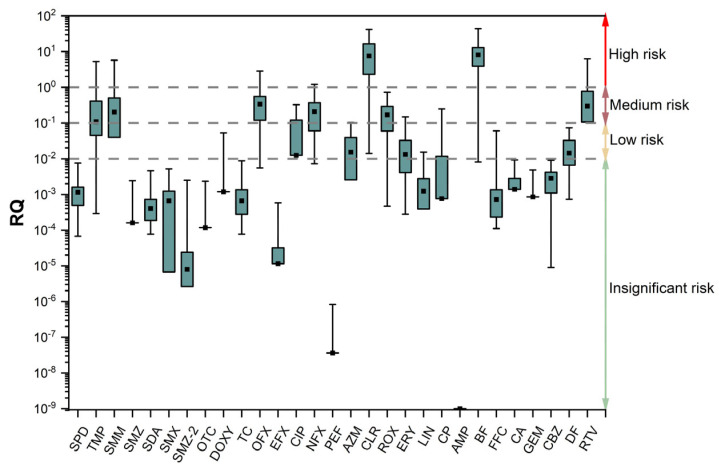
The ecological risks of each pharmaceutical.

Based on the PNEC shown in Table S6. More than 55.2% of the pharmaceuticals investigated in the surface waters of the Beijing North Canal Basin had obvious ecological risks, including low, medium, and high risk (Figure 4). Pharmaceuticals of MLs posed the highest risk rate among the five categories, reaching 100%. SAs have the lowest risk rate, with only two pharmaceuticals posing significant ecological risks, accounting for 28.6%. The risk rates for TCs, FQs, and OTs pharmaceuticals were 33.3%, 60%, and 60%, respectively. TMP, SMM, OFX, NFX, CLR, BF, and RTV have caused high ecological risks in different locations, with CLR and BF causing high ecological risks at over 75% of the sampling sites, with CLR at sampling site S80 in the Qing River posing the most serious ecological risk. Of the pharmaceuticals, 17.2% exhibited medium ecological risk at different sampling sites, except those with high ecological risk. This category includes CIP, AZM, ROX, ERY, and CP, with only ERY causing a medium ecological risk at over 50% of the sampling sites. Pharmaceuticals with low ecological risk, including DOXY, LIN, FFC, and DF, account for 13.8%, with only DF causing low ecological risk at most sampling sites. The remaining research pharmaceuticals do not exhibit significant ecological risks, accounting for 44.8% of the total.

**Figure 5:**
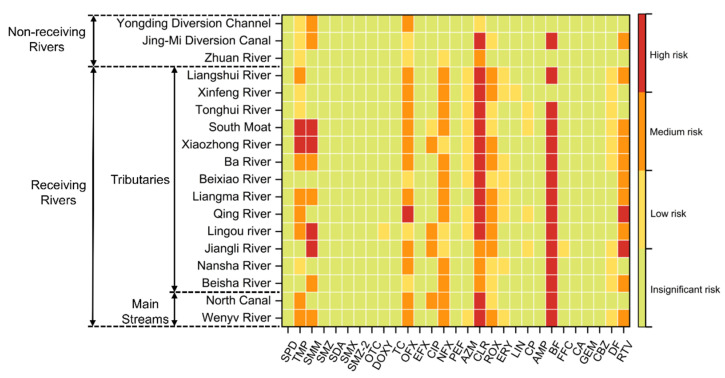
The ecological risks of each pharmaceutical in each river.

The level of ecological risk in all streams of the North Canal Basin was different according to the spatial distribution of pharmaceutical ecological risks (Figure 5). The result showed that more than 55.2% of the pharmaceuticals posed ecological risks in the different studied rivers. TMP, SMM, OFX, CLR, BF, and RTV have all resulted in high ecological risks in different rivers within the study basin, with CLR and BF causing high ecological risks in most rivers. CIP, NFX, and ROX posed moderate ecological risks at their most severe in different rivers. DOXY, AZM, ERY, LIN, CP, FFC, and DF posed only low ecological risks in various rivers, while the remaining pharmaceuticals have not posed any ecological risks. Combining the PNEC of each pharmaceutical, the results showed that high pharmaceutical concentrations and small PNEC values may be responsible for the high ecological risk of pharmaceuticals.

The mean ecological risk rate of each river category was as follows: tributaries (33.7%) > main streams (31.0%) > non-receiving rivers (17.2%), respectively. This shows that a higher mean ecological risk rate occurred in the tributaries and receiving rivers compared to the main streams and non-receiving rivers. This is the same conclusion drawn by Meng et al., who also believe that pollution is more serious in small tributaries than the main streams in the North Canal basin [46]. The result indicates that human discharge behavior is likely to have influenced the level of ecological risk to aquatic organisms. As a major source of discharge in the study basin, controlling discharges from the WWTP may be the most cost-effective measure for dealing with pharmaceutical pollution.

## 4. Conclusions

The study systematically investigated the occurrence, sources, and ecological risks of typical pharmaceuticals in the North Canal Basin. The results showed that pharmaceutical concentrations in the North Canal Basin within Beijing were generally moderate compared to other freshwater study areas, but pharmaceutical pollution was widespread, with the pharmaceutical concentrations ranging from N.D. to 193 ng/L and AMP being detected with a frequency of 100%. Spatially, pharmaceutical concentrations in directly received wastewater rivers are typically higher. In addition, the WWTP effluent and the confluence of village sewage streams may be the primary reason for the sudden increase in total pharmaceutical concentrations at some sampling sites. The sources of pharmaceutical pollution in the study basin are complex, with WWTP effluent contributing to the major pharmaceutical loads, while in suburban areas, a possible contribution of untreated wastewater was demonstrated. In addition, the risk assessment results indicate that the ecological risk widely occurred in the North Canal Basin, with more serious ecological risks being apparent in receiving rivers, which may be related to the fact that some pharmaceuticals cannot be efficiently removed from WWTP. Combined with pollution source analysis, controlling the concentration of pharmaceuticals discharged from the WWTP may be the most cost-effective measure for dealing with pharmaceutical pollution.

The use of active sampling to monitor the occurrence of pharmaceuticals in the environment has a certain contingency, and some of the areas that are difficult to collect from pose challenges. There has been a study of the use of remotely controllable electronic devices instead of manual sampling, and there have also been studies using passive sampling techniques to minimize contingency [47,48,49]. Therefore, we believe that combining passive sampling techniques with electronic devices is a research direction worth exploring.

## Figures and Tables

**Table 1:** The concentration and detection frequency of different pharmaceuticals.

-	Pharmaceuticals	Abbr.	Max (ng/L)	Min (ng/L)	Mean (ng/L)	Detection Frequency (%)
SAs	Sulfapyridine	SPD	75.6	N.D.	12.0	90.2
	Trimethoprim	TMP	153	N.D.	15.6	93.9
	Sulfamonomethoxine	SMM	6.82	N.D.	0.55	53.7
	Sulfamerazine	SMZ	28.8	N.D.	0.86	7.32
	Sulfadiazine	SDA	12.4	N.D.	1.87	76.8
	Sulfamethoxazole	SMX	45.0	N.D.	7.76	73.2
	Sulfadimidine	SMZ-2	75.0	N.D.	1.79	57.3
TCs	Oxytetracycline	OTC	17.7	N.D.	0.54	11.0
	Doxycycline hyclate	DOXY	106	N.D.	3.92	20.7
	Tetracycline	TC	66.2	N.D.	9.34	79.3
FQs	Ofloxacin	OFX	114	<0.16	18.7	100
	Enrofloxacin	EFX	18.7	N.D.	0.97	31.7
	Ciprofloxacin	CIP	13.0	N.D.	2.14	31.7
	Norfloxacin	NFX	193	N.D.	40.0	90.2
	Pefloxacin	PEF	7.63	N.D.	0.20	4.88
MLs	Azithromycin	AZM	49.5	N.D.	11.8	67.1
	Clarithromycin	CLR	83.2	N.D.	20.9	98.8
	Roxithromycin	ROX	47.8	N.D.	12.7	97.6
	Erythromycin	ERY	29.6	N.D.	4.42	86.6
OTs	Lincomycin hydrochloride	LIN	45.8	N.D.	5.74	62.2
	Chloramphenicol	CP	32.3	N.D.	1.18	47.6
	Ampicillin	AMP	N.D.	N.D.	0.00	0.00
	Bezafibrate	BF	29.4	N.D.	6.56	90.2
	Florfenicol	FFC	139	N.D.	3.55	76.8
	Clofibric acid	CA	25.4	N.D.	3.18	26.8
	Gemfibrozil	GEM	14.8	N.D.	0.48	4.88
	Carbamazepine	CBZ	18.7	N.D.	6.02	91.5
	Diclofenac	DF	54.8	N.D.	15.3	92.7
	Ritonavir	RTV	18.0	N.D.	1.64	59.8

N.D.: not detected.

## Data Availability

Data are contained within the article and Supplementary Materials.

## Supplementary Materials

The following supporting information can be downloaded at https://www.mdpi.com/article/10.3390/toxics12030171/s1: Table S1. Related information for target pharmaceuticals; Table S2. Gradient elution procedure; Table S3. Standard curve and MDL; Table S4. Selection method of assessment factor; Table S5. Comparison of pharmaceutical concentrations between the North Canal Basin and other study areas [16,42,50,51,52,53,54,55,56,57,58,59,60,61,62,63,64,65,66]; Table S6. PNEC values of pharmaceuticals and calculated method; Table S7. List of abbreviations used in the manuscript except pharmaceuticals; Figure S1. Interpolation map of inverse distance weights of each pharmaceutical concentration category in the North Canal Basin; Figure S2. The contributions of all the identified sources to the pharmaceuticals in the North Canal Basin; Figure S3. Interpolation map of inverse distance weights of each pharmaceutical concentration factor in the North Canal Basin.

## References

[1] Bavumiragira J.P., Ge J., Yin H. (2022). Fate and Transport of Pharmaceuticals in Water Systems: A Processes Review. Sci. Total Environ..

[2] Zhang Q.-Q., Ying G.-G., Pan C.-G., Liu Y.-S., Zhao J.-L. (2015). Comprehensive Evaluation of Antibiotics Emission and Fate in the River Basins of China: Source Analysis, Multimedia Modeling, and Linkage to Bacterial Resistance. Environ. Sci. Technol..

[3] Anh H.Q., Le T.P.Q., Da Le N., Lu X.X., Duong T.T., Garnier J., Rochelle-Newall E., Zhang S., Oh N.-H., Oeurng C. (2021). Antibiotics in Surface Water of East and Southeast Asian Countries: A Focused Review on Contamination Status, Pollution Sources, Potential Risks, and Future Perspectives. Sci. Total Environ..

[4] Ebele A.J., Abou-Elwafa Abdallah M., Harrad S. (2017). Pharmaceuticals and Personal Care Products (PPCPs) in the Freshwater Aquatic Environment. Emerg. Contam..

[5] Pandey R.P., Yousef A.F., Alsafar H., Hasan S.W. (2023). Surveillance, Distribution, and Treatment Methods of Antimicrobial Resistance in Water: A Review. Sci. Total Environ..

[6] García-Valverde M., Aragonés A.M., Andújar J.A.S., García M.D.G., Martínez-Bueno M.J., Fernández-Alba A.R. (2023). Long-Term Effects on the Agroecosystem of Using Reclaimed Water on Commercial Crops. Sci. Total Environ..

[7] Pu S., Shao Z., Yang L., Liu R., Bi J., Ma Z. (2019). How Much Will the Chinese Public Pay for Air Pollution Mitigation? A Nationwide Empirical Study Based on a Willingness-to-Pay Scenario and Air Purifier Costs. J. Clean. Prod..

[8] K’oreje K.O., Okoth M., Van Langenhove H., Demeestere K. (2020). Occurrence and Treatment of Contaminants of Emerging Concern in the African Aquatic Environment: Literature Review and a Look Ahead. J. Environ. Manag..

[9] Chaves M.D.J.S., Kulzer J., Pujol De Lima P.D.R., Barbosa S.C., Primel E.G. (2022). Updated Knowledge, Partitioning and Ecological Risk of Pharmaceuticals and Personal Care Products in Global Aquatic Environments. Environ. Sci. Processes Impacts.

[10] Yu Y., Wang S., Yu P., Wang D., Hu B., Zheng P., Zhang M. (2024). A Bibliometric Analysis of Emerging Contaminants (ECs) (2001−2021): Evolution of Hotspots and Research Trends. Sci. Total Environ..

[11] Li Y., Zhang L., Ding J., Liu X. (2020). Prioritization of Pharmaceuticals in Water Environment in China Based on Environmental Criteria and Risk Analysis of Top-Priority Pharmaceuticals. J. Environ. Manag..

[12] Li Q., Bu Q., Cao H., Hong C., Wu X., Guo Y., Jiang W. (2023). Simultaneous Determination of 33 Pharmaceuticals in Surface Water Using Solid-Phase Extraction and Liquid Chromatography-Tandem Mass Spectrometry. Environ. Monit. China.

[13] Paatero P., Tapper U. (1994). Positive Matrix Factorization: A Non-negative Factor Model with Optimal Utilization of Error Estimates of Data Values. Environmetrics.

[14] Men C., Liu R., Wang Q., Guo L., Miao Y., Shen Z. (2019). Uncertainty Analysis in Source Apportionment of Heavy Metals in Road Dust Based on Positive Matrix Factorization Model and Geographic Information System. Sci. Total Environ..

[15] Wang L., Wang Y., Li H., Zhu Y., Liu R. (2022). Occurrence, Source Apportionment and Source-Specific Risk Assessment of Antibiotics in a Typical Tributary of the Yellow River Basin. J. Environ. Manag..

[16] Wu S., Hua P., Gui D., Zhang J., Ying G., Krebs P. (2022). Occurrences, Transport Drivers, and Risk Assessments of Antibiotics in Typical Oasis Surface and Groundwater. Water Res..

[17] Chinese Research Academy of Environmental Sciences, Research Center for Eco-Environmental Sciences, Chinese Academy of Sciences, National Marine Environmental Monitoring Center, China National Environmental Monitoring Centre (2022). Technical Guideline for Deriving Water Quality Criteria for Freshwater Organisms.

[18] Van Leeuwen K. (2003). Technical Guidance Documenton Risk Assessment.

[19] Kuroda K., Li C., Dhangar K., Kumar M. (2021). Predicted Occurrence, Ecotoxicological Risk and Environmentally Acquired Resistance of Antiviral Drugs Associated with COVID-19 in Environmental Waters. Sci. Total Environ..

[20] Duan L., Zhang Y., Wang B., Cagnetta G., Deng S., Huang J., Wang Y., Yu G. (2020). Characteristics of Pharmaceutically Active Compounds in Surface Water in Beijing, China: Occurrence, Spatial Distribution and Biennial Variation from 2013 to 2017. Environ. Pollut..

[21] Yuan X., Hu J., Li S., Yu M. (2020). Occurrence, Fate, and Mass Balance of Selected Pharmaceutical and Personal Care Products (PPCPs) in an Urbanized River. Environ. Pollut..

[22] Li J., Li W., Liu K., Guo Y., Ding C., Han J., Li P. (2022). Global Review of Macrolide Antibiotics in the Aquatic Environment: Sources, Occurrence, Fate, Ecotoxicity, and Risk Assessment. J. Hazard. Mater..

[23] Mehrtens A., Licha T., Burke V. (2021). Occurrence, Effects and Behaviour of the Antibiotic Lincomycin in the Agricultural and Aquatic Environment—A Review. Sci. Total Environ..

[24] Wang H., Tang C., Wang Y., Han M., Jiang F., Jiang L., Wu J., Fu C., Chen Y., Jiang Q. (2021). Urinary Antibiotic Level of School Children in Shanghai, East China, 2017–2020. Environ. Pollut..

[25] Duan L., Zhang Y., Wang B., Yu G., Gao J., Cagnetta G., Huang C., Zhai N. (2022). Wastewater Surveillance for 168 Pharmaceuticals and Metabolites in a WWTP: Occurrence, Temporal Variations and Feasibility of Metabolic Biomarkers for Intake Estimation. Water Res..

[26] Zhang Y., Wang B., Cagnetta G., Duan L., Yang J., Deng S., Huang J., Wang Y., Yu G. (2018). Typical Pharmaceuticals in Major WWTPs in Beijing, China: Occurrence, Load Pattern and Calculation Reliability. Water Res..

[27] Zhang X., Zhao H., Du J., Qu Y., Shen C., Tan F., Chen J., Quan X. (2017). Occurrence, Removal, and Risk Assessment of Antibiotics in 12 Wastewater Treatment Plants from Dalian, China. Environ. Sci. Pollut. Res..

[28] Yuan X., Qiang Z., Ben W., Zhu B., Qu J. (2015). Distribution, Mass Load and Environmental Impact of Multiple-Class Pharmaceuticals in Conventional and Upgraded Municipal Wastewater Treatment Plants in East China. Environ. Sci. Processes Impacts.

[29] Prabhasankar V.P., Joshua D.I., Balakrishna K., Siddiqui I.F., Taniyasu S., Yamashita N., Kannan K., Akiba M., Praveenkumarreddy Y., Guruge K.S. (2016). Removal Rates of Antibiotics in Four Sewage Treatment Plants in South India. Environ. Sci. Pollut. Res..

[30] Liu X., Zhang G., Liu Y., Lu S., Qin P., Guo X., Bi B., Wang L., Xi B., Wu F. (2019). Occurrence and Fate of Antibiotics and Antibiotic Resistance Genes in Typical Urban Water of Beijing, China. Environ. Pollut..

[31] Byzova N.A., Smirnova N.I., Zherdev A.V., Eremin S.A., Shanin I.A., Lei H.-T., Sun Y., Dzantiev B.B. (2014). Rapid Immunochromatographic Assay for Ofloxacin in Animal Original Foodstuffs Using Native Antisera Labeled by Colloidal Gold. Talanta.

[32] Shi Y., Liu J., Zhuo L., Yan X., Cai F., Luo W., Ren M., Liu Q., Yu Y. (2020). Antibiotics in Wastewater from Multiple Sources and Surface Water of the Yangtze River in Chongqing in China. Environ. Monit. Assess..

[33] Cai S., Liu Y., Chen J. (2020). FeCu-Biochar Enhances the Removal of Antibacterial Sulfapyridine from Groundwater by Activation of Persulfate. Environ. Chem. Lett..

[34] Carneiro R.B., Sabatini C.A., Santos-Neto Á.J., Zaiat M. (2019). Feasibility of Anaerobic Packed and Structured-Bed Reactors for Sulfamethoxazole and Ciprofloxacin Removal from Domestic Sewage. Sci. Total Environ..

[35] Qiu T., Liu L., Gao M., Zhang L., Tursun H., Wang X. (2016). Effects of Solid-Phase Denitrification on the Nitrate Removal and Bacterial Community Structure in Recirculating Aquaculture System. Biodegradation.

[36] Wang J., Wang S. (2018). Microbial Degradation of Sulfamethoxazole in the Environment. Appl. Microbiol. Biotechnol..

[37] De Smet J., Boyen F., Croubels S., Rasschaert G., Haesebrouck F., De Backer P., Devreese M. (2018). Similar Gastro-Intestinal Exposure to Florfenicol after Oral or Intramuscular Administration in Pigs, Leading to Resistance Selection in Commensal *Escherichia coli*. Front. Pharmacol..

[38] Bagnis S., Boxall A., Gachanja A., Fitzsimons M., Murigi M., Snape J., Tappin A., Wilkinson J., Comber S. (2020). Characterization of the Nairobi River Catchment Impact Zone and Occurrence of Pharmaceuticals: Implications for an Impact Zone Inclusive Environmental Risk Assessment. Sci. Total Environ..

[39] Kondor A.C., Molnár É., Jakab G., Vancsik A., Filep T., Szeberényi J., Szabó L., Maász G., Pirger Z., Weiperth A. (2022). Pharmaceuticals in Water and Sediment of Small Streams under the Pressure of Urbanization: Concentrations, Interactions, and Risks. Sci. Total Environ..

[40] Bernus I., Dickinson R.G., Hooper W.D., Eadie M.J. (1994). Early Stage Autoinduction of Carbamazepine Metabolism in Humans. Eur. J. Clin. Pharmacol..

[41] Sawchuk R.J., Maloney J.A., Cartier L.L., Rackley R.J., Chan K.K.H., Lau H.S.L. (1995). Analysis of Diclofenac and Four of Its Metabolites in Human Urine by HPLC. Pharm. Res..

[42] Zhang Z., Zhou Y., Han L., Guo X., Wu Z., Fang J., Hou B., Cai Y., Jiang J., Yang Z. (2022). Impacts of COVID-19 Pandemic on the Aquatic Environment Associated with Disinfection Byproducts and Pharmaceuticals. Sci. Total Environ..

[43] Beijing Municipal Health Commission Beijing Statistical Bulletin on the Development of Health Undertakings in 2021. https://wjw.beijing.gov.cn/sjfb/bjstjgb/bjstjgb2021/202306/t20230614_3133785.html.

[44] Dai G., Wang B., Fu C., Dong R., Huang J., Deng S., Wang Y., Yu G. (2016). Pharmaceuticals and Personal Care Products (PPCPs) in Urban and Suburban Rivers of Beijing, China: Occurrence, Source Apportionment and Potential Ecological Risk. Environ. Sci. Processes Impacts.

[45] Wu Y., Song S., Chen X., Shi Y., Cui H., Liu Y., Yang S. (2023). Source-Specific Ecological Risks and Critical Source Identification of PPCPs in Surface Water: Comparing Urban and Rural Areas. Sci. Total Environ..

[46] Meng Y., Zhang J., Fiedler H., Liu W., Pan T., Cao Z., Zhang T. (2022). Influence of Land Use Type and Urbanization Level on the Distribution of Pharmaceuticals and Personal Care Products and Risk Assessment in Beiyun River, China. Chemosphere.

[47] Zhang J., Huang W., Wu R., Yan Z., Tan G., Zhu C., Gao W., Hu B. (2023). Real-Time and Online Monitoring of Hazardous Volatile Organic Compounds in Environmental Water by an Unmanned Shipborne Mass Spectrometer System. Environ. Sci. Technol..

[48] Cao H., Bu Q., Li Q., Gao X., Xie H., Gong W., Wang X., Yang L., Tang J. (2022). Development and Applications of Diffusive Gradients in Thin Films for Monitoring Pharmaceuticals in Surface Waters. Environ. Pollut..

[49] Yi J., Huang X., Hou J., Xiong J., Qian Z., Liu S., Zhang J., Yin D., Li J., Su Q. (2023). Occurrence and Distribution of PPCPs in Water from Two Largest Urban Lakes of China: First Perspective from DGT in-Situ Measurement. Sci. Total Environ..

[50] Gao H., Zhao F., Li R., Jin S., Zhang H., Zhang K., Li S., Shu Q., Na G. (2022). Occurrence and Distribution of Antibiotics and Antibiotic Resistance Genes in Water of Liaohe River Basin, China. J. Environ. Chem. Eng..

[51] Linghu K., Wu Q., Zhang J., Wang Z., Zeng J., Gao S. (2023). Occurrence, Distribution and Ecological Risk Assessment of Antibiotics in Nanming River: Contribution from Wastewater Treatment Plant and Implications of Urban River Syndrome. Process Saf. Environ. Prot..

[52] Guo F., Wang Y., Peng J., Huang H., Tu X., Zhao H., Zhan N., Rao Z., Zhao G., Yang H. (2022). Occurrence, Distribution, and Risk Assessment of Antibiotics in the Aquatic Environment of the Karst Plateau Wetland of Yangtze River Basin, Southwestern China. Int. J. Environ. Res. Public Health.

[53] Ci M., Zhang G., Yan X., Dong W., Xu W., Wang W., Fan Y. (2021). Occurrence of Antibiotics in the Xiaoqing River Basin and Antibiotic Source Contribution-a Case Study of Jinan City, China. Environ. Sci. Pollut. Res..

[54] Zhou Q., Liu G., Arif M., Shi X., Wang S. (2022). Occurrence and Risk Assessment of Antibiotics in the Surface Water of Chaohu Lake and Its Tributaries in China. Sci. Total Environ..

[55] Malnes D., Ahrens L., Köhler S., Forsberg M., Golovko O. (2022). Occurrence and Mass Flows of Contaminants of Emerging Concern (CECs) in Sweden’s Three Largest Lakes and Associated Rivers. Chemosphere.

[56] Deng W.-J., Li N., Ying G.-G. (2018). Antibiotic Distribution, Risk Assessment, and Microbial Diversity in River Water and Sediment in Hong Kong. Environ. Geochem. Health.

[57] Ding H., Wu Y., Zhang W., Zhong J., Lou Q., Yang P., Fang Y. (2017). Occurrence, Distribution, and Risk Assessment of Antibiotics in the Surface Water of Poyang Lake, the Largest Freshwater Lake in China. Chemosphere.

[58] Liu Y., Chen Y., Feng M., Chen J., Shen W., Zhang S. (2021). Occurrence of Antibiotics and Antibiotic Resistance Genes and Their Correlations in River-Type Drinking Water Source, China. Environ. Sci. Pollut. Res..

[59] Yu X., Yu F., Li Z., Zhan J. (2023). Occurrence, Distribution, and Ecological Risk Assessment of Pharmaceuticals and Personal Care Products in the Surface Water of the Middle and Lower Reaches of the Yellow River (Henan Section). J. Hazard. Mater..

[60] Guo X., Song R., Lu S., Liu X., Chen J., Wan Z., Bi B. (2022). Multi-Media Occurrence of Antibiotics and Antibiotic Resistance Genes in East Dongting Lake. Front. Environ. Sci..

[61] Zou S., Huang F., Chen L., Liu F. (2018). The Occurrence and Distribution of Antibiotics in the Karst River System in Kaiyang, Southwest China. Water Supply.

[62] Gopal C.M., Bhat K., Ramaswamy B.R., Kumar V., Singhal R.K., Basu H., Udayashankar H.N., Vasantharaju S.G., Praveenkumarreddy Y., Shailesh (2021). Seasonal Occurrence and Risk Assessment of Pharmaceutical and Personal Care Products in Bengaluru Rivers and Lakes, India. J. Environ. Chem. Eng..

[63] Feng J., Liu Q., Ru X., Xi N., Sun J. (2020). Occurrence and Distribution of Priority Pharmaceuticals in the Yellow River and the Huai River in Henan, China. Environ. Sci. Pollut. Res..

[64] Corrêa J.M.M., Sanson A.L., Machado C.F., Aquino S.F., Afonso R.J.C.F. (2021). Occurrence of Contaminants of Emerging Concern in Surface Waters from Paraopeba River Basin in Brazil: Seasonal Changes and Risk Assessment. Environ. Sci. Pollut. Res..

[65] Tang J., Sun J., Wang W., Yang L., Xu Y. (2021). Pharmaceuticals in Two Watersheds in Eastern China and Their Ecological Risks. Environ. Pollut..

[66] Domínguez-García P., Rodríguez R.R., Barata C., Gómez-Canela C. (2023). Presence and Toxicity of Drugs Used to Treat SARS-CoV-2 in Llobregat River, Catalonia, Spain. Environ. Sci. Pollut. Res..

