# Supplementary material for: Occurrence, Source Apportionment, and Ecological Risk of Typical Pharmaceuticals in Surface Waters of Beijing, China
Source: Toxics. 2024 Feb 23;12(3):171. doi: 10.3390/toxics12030171 (PMC10975916; doi:10.3390/toxics12030171)
Supplement: Supplementary file 1 [file toxics-12-00171-s001.zip › toxics-2876086-supplementary.pdf]

Supplementary Material for

Occurrence, Source Apportionment, and Ecological Risk of Typical Pharmaceuticals in Surface Waters of Beijing, China

Yonghao Huangfu [1], Qingshan Li [1], Weiwei Yang [1], Qingwei Bu [1,*], Lei Yang [2], Jianfeng Tang [3], Jie Gan [4,*]

[1] School of Chemical & Environmental Engineering, China University of Mining & Technology-Beijing, Beijing 100083, P.R. China

[2] State Key Laboratory of Urban and Regional Ecology, Research Center for Eco-Environmental Science, Chinese Academy of Sciences, Beijing 100085, P.R. China

[3] Key Laboratory of Urban Environment and Health, Institute of Urban Environment, Chinese Academy of Sciences, Xiamen 361021, P.R. China

[4] Hunan Ecological and Environmental Monitoring Center, Changsha 410014, P.R. China

*** Corresponding author**

Address correspondence to Qingwei Bu, E-mail: qingwei.bu@cumtb.edu.cn

Address correspondence to Jie Gan, E-mail: ganjie1982@163.com

Contents

Study sites

Sample pretreatment

Instrumental analysis

Table S1. Related information for target pharmaceuticals.

Table S2. Gradient elution procedure.

Table S3. Standard curve and MDL.

Table S4. Selection method of assessment factor.

Table S5. Comparison of pharmaceutical concentrations between the North Canal Basin and other study areas.

Table S6. PNEC values of Pharmaceuticals and calculated method.

Figure S1. Interpolation map of inverse distance weights of each pharmaceutical concentration category in the North Canal Basin.

Figure S2. The contributions of all the identified sources to the pharmaceuticals in the North Canal Basin.

Figure S3. Interpolation map of inverse distance weights of each pharmaceutical concentration factor in the North Canal Basin.

Study areas and sites

The North Canal is the only water system that originates in Beijing and is the most important river flowing through downtown Beijing. The population within its basin accounts for over 70% of the city population, contributing to more than 80% of Beijing's gross domestic product (GDP). It is the most populous basin in Beijing, characterized by the highest industrial concentration and urbanization level. Situated in the northwest of the North China Plain, the North Canal Basin serves as the transitional region between the plain, plateau, and mountainous terrains, featuring a typical temperate continental climate. The North Canal Basin encompasses the main stem of the North Canal and Wen Yu River, along with five major tributaries: Sha River, Qing River, Ba River, Tonghui River, and Liangshui River. In addition, numerous smaller tributaries cover the urban and agricultural areas of Beijing, Tianjin, and Hebei. The total basin area is 4,423 km^2.

The rules for selecting sampling sites are as follows: First, the target rivers were selected before setting up the sampling points. The main stream of Beijing's North Canal basin and five major tributaries, as well as other rivers that play an important role in people's production and life in Beijing, were selected, and sampling points were set up at locations where there might be significant changes in pharmaceutical concentrations, such as wastewater treatment plant effluents and river confluence.

In this study, the receiving rivers of the North Canal Basin are categorized into tributaries (Beisha River, Nansha River, Lingou River, Jiangli River, Qing River, Ba River, Tonghui River, Beixiao River, Liangma River, South Moat River, Xiaozhong River, Liangshui River, and Xinfeng River) and main streams (Wenyv river and North Canal). In addition, the non-receiving rivers include the Jing-Mi Diversion Channel, Yongding Diversion Channel, and Zhuan River.

Sample pretreatment

To reduce the biological activity in the collected water, the samples were

transferred to the laboratory at 4° C and immediately filtered through the 0.45 μm glass fiber filter membrane. Pre-experimentation showed that different pharmaceuticals had different recoveries under different acid–base conditions. Two 5L filtered water samples were measured, and the pH was adjusted at 3 and 9 using HCL and NaOH, respectively. A total of 10 μL of the internal standard solution (concentration: 10 μg/mL) was added to each sample. The samples were extracted via solid phase extraction (SPE), which was activated by methanol and ultrapure water. Samples were enriched at a flow rate of 4 to 8 mL/min through the SPE column under vacuum pumping, followed by the column being rinsed with 5 mL of ultrapure water and then blow dry. The sample was eluted with 8 mL of methanol into a KD concentrator. The eluate was blown to 0.5 mL in a gentle stream of nitrogen. The sample solution was reconstituted with methanol and ultrapure water (V: V=1:9), vortexed until well mixed, and analyzed immediately.

Instrumental analysis

Determination of the target analytes was carried out using a high-performance liquid chromatograph–triple quadrupole mass spectrometer system (LCMS-8040, Shimadzu, Japan). Separation was performed on a Shim-pack XR-ODS reversed-phase column (2 mm×75 mm, 2.2 μm), the temperature was maintained at 30 °C. 0.2% formic acid-2 mmol/L ammonium acetate aqueous solution, and acetonitrile were used as mobile phase A and mobile phase B for gradient elution, respectively. The gradient elution conditions are described in Table S2. The sample injection volume was 10 μL. Multiple reaction monitoring (MRM) was used for the detection and quantification of precursor and product ions.

Table S1. Related information for target pharmaceuticals.

Categories	Abbr. of Categories	Pharmaceuticals	Abbr. of Pharmaceuticals	CAS Number	Precursor ion (m/z)	Product ions (m/z)	Collision Energy (V)	Retention Time (min)
Sulfonamides	SAs	Sulfapyridine	SPD	144-83-2	250.1	156.00*, 92.10	-16, -28	3.815
		Trimethoprim	TMP	738-70-5	291.2	230.10*, 261.05	-24, -35	4.271
		Sulfamonomethoxine	SMM	1220-83-3	281.1	155.90*, 92.10	-18, -30	8.834
		Sulfamerazine	SMZ	127-79-7	265.1	156.10*, 92.10	-17, -18	4.290
		Sulfadiazine	SDA	68-35-9	251.1	156.00*, 92.10	-16, -28	2.878
		Sulfamethoxazole	SMX	723-46-6	253.9	156.00*, 92.10	-15, -28	9.957
		Sulfadimidine	SMZ-2	57-68-1	278.9	186.05*, 92.15	-17, -25	5.912
Tetracyclines	TCs	Oxytetracycline	OTC	79-57-2	461.2	426.15*, 442.90	-19, -13	5.613
		Doxycycline hyclate	DOXY	24390-14-5	445.2	428.15*, 413.30	-19, -34	10.455
		Tetracycline	TC	60-54-8	445.2	410.10*, 427.10	-20, -14	6.562
Fluoroquinolones	FQs	Ofloxacin	OFX	82419-36-1	362.1	318.25*, 261.10	-20, -30	6.215
		Enrofloxacin	EFX	93106-60-6	360.2	316.10*, 342.20	-20, -22	8.117
		Ciprofloxacin	CIP	85721-33-1	332.2	314.15*, 231.00	-22, -18	6.870
		Norfloxacin	NFX	70458-96-7	320.2	302.10*, 230.95	-21, -17	6.310
		Pefloxacin	PEF	70458-92-3	334.2	316.15*, 302.15	-19, -19	6.587
Macrolides	MLs	Azithromycin	AZM	83905-01-5	749.6	158.05*, 591.40	-31, -43	12.708
		Clarithromycin	CLR	81103-11-9	748.4	158.15*, 590.40	-34, -21	12.699
		Roxithromycin	ROX	80214-83-1	837.4	158.10*, 679.30	-39, -24	12.810
		Erythromycin	ERY	114-07-8	734.3	158.15*, 576.30	-35, -21	11.645
Others	OTs	Lincomycin hydrochloride	LIN	859-18-7	407.4	126.10*, 359.10	-29, -19	2.793
		Chloramphenicol	CP	56-75-7	321.0	152.15*, 257.15	17, 11	11.130
		Ampicillin	AMP	69-53-4	350.1	106.15*, 160.00	-21, -17	4.353
		Bezafibrate	BF	41859-67-0	360.2	274.15*, 154.10	18, 30	14.582
		Florfenicol	FFC	73231-34-2	356.1	336.05*, 185.10	9, 20	10.105
		Clofibric acid	CA	882-09-7	213.0	127.05*	14	14.402
		Gemfibrozil	GEM	25812-30-0	249.2	121.20*, 113.10	16	16.463
		Carbamazepine	CBZ	298-46-4	237.0	194.05*, 192.10	-19, -21	12.887
		Diclofenac	DF	15307-86-5	294.0	250.10*, 214.00	12, 21	15.768
		Ritonavir	RTV	155213-67-5	721.3	296.10*, 268.05	-19, -30	15.960
Internal standards	-	Roxithromycin-d7	ROX-D7	80214-83-1	844.7	158.15*, 686.50	-35, -25	12.773
		Chloramphenicol-d5	CP-D5	202480-68-0	326.0	157.20*, 262.30	17, 12	11.101
		Sulfamethazine-d4	SMT-D4	1020719-82-7	283.2	186.10*, 124.25	-17, -25	5.806
		Carbamazepine-d10	CBZ-D5	132183-78-9	247.2	204.15*, 201.00	-21, -26	12.807
		Norfloxacin-d5	NFX-D5	1015856-57-1	325.3	307.20*, 231.15	-20, -42	6.213
		Demeclocycline	DMC	127-33-3	465.2	448.05*, 430.00	-19, -21	8.445

Table S2. Gradient elution procedure.

	Time（min）	Initial	5.00	7.00	11.00	14.00	16.00	18.00	18.01	22.00
Mobile phase A	0.2% formic acid-2 mmol/L ammonium acetate aqueous solution	100	100	20	20	2	2	100	100	Stop
Mobile phase B	Acetonitrile（%）	10	15	20	40	60	95	95	10	Stop

Table S3. Standard curve and MDL.

Pharmaceuticals	Standard Curve Equation	Linearity Range (ng/mL)	R^2	MDL (ng/L)
SPD	y=0.9731x-0.0509	0.1~200	0.9994	1.34
TMP	y=2.5510x+0.1148	0.05~200	0.9972	0.02
SMM	y=0.4180x+0.0158	0.2~200	0.9986	0.10
SMZ	y=0.5302x-0.0120	0.05~200	0.9986	3.83
SDA	y=0.2967x-0.0067	0.05~200	0.9976	0.42
SMX	y=0.5125x-0.2358	0.05~200	0.9962	0.12
SMZ2	y=24.4990x-1.5872	0.05~150	0.9947	0.16
OTC	y=0.9986x-0.1269	0.05~200	0.9987	1.78
DOXY	y=1.9541x+0.2491	0.1~200	0.9967	4.76
TC	y=2.0257x-0.2082	1~150	0.9965	1.16
OFX	y=6.3641x+0.0271	0.1~150	0.9990	0.16
EFX	y=6.4875x+0.2136	0.05~200	0.9963	0.73
CIP	y=1.0943x+0.0012	5~150	0.9985	0.99
NFX	y=0.8113x-0.0245	0.05~200	0.9975	2.32
PEF	y=2.0606x-0.0965	0.1~200	0.9976	0.67
AZM	y=0.1972x+0.0055	0.05~200	0.9985	2.47
CLR	y=1.6096x+0.0561	0.05~200	0.9984	0.06
ROX	y=1.1006x+0.0329	0.05~200	0.9977	0.06
ERY	y=0.0231x-0.0005	0.05~200	0.9974	0.11
LIN	y=0.0567x-0.0013	0.05~200	0.9976	2.33
CP	y=0.7677x+0.0223	1~200	0.9989	0.20
AMP	y=3.2100x-1.0503	0.05~200	0.9952	1.79
BF	y=0.0277x-0.0001	0.05~200	0.9988	0.01
FFC	y=1.1537x+0.0749	1~200	0.9955	0.51
CA	y=0.0231x-0.0005	0.5~200	0.9974	7.60
GEM	y=0.0006x-0.0002	0.05~200	0.9985	5.23
CBZ	y=1.0033x+0.0152	0.05~200	0.9993	0.04
DF	y=0.5602x+0.0237	0.05~200	0.9987	1.09
RTV	y=121.426x-1.5162	0.05~200	0.9971	0.62

Table S4. Selection method of assessment factor.

Available data	Assessment factor
At least one short-term $L(E)C_{50}$ from each of three trophic levels of the base set (fish, Daphnia, and algae)	1000
One long-term NOEC (either fish or Daphnia)	100
Two long-term NOECs from species representing two trophic levels (fish and/or Daphnia and/or algae)	50
Long-term NOECs from at least three species (normally fish, Daphnia, and algae) representing three trophic levels	10
Species sensitivity distribution (SSD) method	5-1<br>(to be fully justified case by case)
Field data or model ecosystems	Reviewed on a case-by-case basis

Table S5. Comparison of pharmaceutical concentrations between the North Canal basin and other study areas.

Pharmaceuticals	Abb	Max (ng/L)	Min (ng/L)	Mean (ng/L)	DF (%)	Region	Reference
**Sulfonamides**	**SAs**						
Sulfapyridine	SPD	75.6	< MDL	12.0	90.2	North Canal	Present study
		1.31	N.D.	0.08	6.90	Liao River	[1]
		19.3	N.D.	1.1	15.4	Nanming River	[2]
		2.30	N.D.	0.1	5.00	Yangtze River	[3]
Trimethoprim	TMP	153	N.D.	15.6	93.9	North Canal	Present study
		62.9	N.D.	12.9	79.4	Nanming River	[2]
		63.6	< MDL	25.6	100	Xiaoqing River	[4]
		57.2	N.D.	6.55	27.0	Chao Lake	[5]
		2.2	-	0.46	51.0	Lake Vanern, Lake Vattern, and Lake Malaren	[6]
Sulfamonomethoxine	SMM	6.82	N.D.	0.55	53.7	North Canal	Present study
		N.D.	N.D.	N.D.	0	Liao River	[1]
		4.30	N.D.	0.50	35.0	Yangtze River	[3]
		N.D.	N.D.	N.D.	0.00	Sangong River	[7]
Sulfamerazine	SMZ	28.8	N.D.	0.86	7.32	North Canal	Present study
		4.63	N.D.	0.41	24.1	Liao River	[1]
		< MDL	N.D.	N.D.	33.0	Xiaoqing River	[4]
Sulfadiazine	SDA	12.4	N.D.	1.87	76.8	North Canal	Present study
		2.15	N.D.	0.53	37.9	Liao River	[1]
		1.60	N.D.	0.10	2.90	Nanming River	[2]
		9.51	N.D.	2.46	87.0	Xiaoqing River	[4]
Sulfamethoxazole	SMX	45.0	N.D.	7.76	73.2	North Canal	Present study
		14.8	N.D.	3.61	65.5	Liao River	[1]
		290	N.D.	11.9	58.8	Nanming River	[2]
		190	N.D.	74.4	93.0	Xiaoqing River	[4]
		12.0	-	2.70	61.0	Lake Vanern, Lake Vattern, and Lake Malaren	[6]
Sulfadimidine	SMZ-2	75.0	N.D.	1.79	57.3	North Canal	Present study
		2.30	N.D.	0.40	24.1	Liao River	[1]
		29.9	N.D.	-	76.9	River in Hong Kong	[8]
		7.7	N.D.	-	87.5	Poyang Lake	[9]
**Tetracyclines**	**TCs**						
Oxytetracycline	OTC	17.7	N.D.	0.54	11.0	North Canal	Present study
		N.D.	N.D.	N.D.	0	Xiaoqing River	[4]
		76.7	N.D.	4.80	11.1	Nanming River	[2]
		18.0	N.D.	0.62	3.45	Liao River	[1]
Doxycycline hyclate	DOXY	106	N.D.	3.92	20.7	North Canal	Present study
		57.9	N.D.	6.01	34.5	Liao River	[1]
		1.90	0.30	0.60	50.0	Yangtze River	[3]
		0.87	N.D.	0.39	88.9	Sangong River	[7]
Tetracycline	TC	66.2	N.D.	9.34	79.3	North Canal	Present study
		N.D.	N.D.	N.D.	0	Xiaoqing River	[4]
		157	N.D.	21.4	25.0	Chao Lake	[5]
		25.5	N.D.	1.9	7.70	Nanming River	[2]
**Fluoroquinolones**	**FQs**						
Ofloxacin	OFX	114	<0.16	18.7	100	North Canal	Present study
		339	7.31	79.0	100	Xiaoqing River	[4]
		55.1	N.D.	4.55	11.0	Chao Lake	[5]
		645	N.D.	137	91.2	Nanming River	[2]
Enrofloxacin	EFX	18.7	N.D.	0.97	31.7	North Canal	Present study
		1.18	N.D.	< MDL	53.0	Xiaoqing River	[4]
		5.47	N.D.	0.35	13.8	Liao River	[1]
		5.43	3.00	4.31	100	Yangtze River	[10]
Ciprofloxacin	CIP	13.0	N.D.	2.14	31.7	North Canal	Present study
		N.D.	N.D.	N.D.	0	Xiaoqing River	[4]
		187	N.D.	13.5	23.0	Chao Lake	[5]
		5.02	N.D.	0.17	3.45	Liao River	[1]

Pharmaceuticals	Abb	Max (ng/L)	Min (ng/L)	Mean (ng/L)	DF (%)	Region	Reference
Norfloxacin	NFX	193	N.D.	40.0	90.2	North Canal	Present study
		285	N.D.	36.6	61.8	Nanming River	[2]
		13.6	N.D.	0.65	6.90	Liao River	[1]
		N.D.	N.D.	N.D.	0	Yangtze River	[10]
Pefloxacin	PEF	7.63	N.D.	0.20	4.88	North Canal	Present study
		5.00	N.D.	0.36	7.14	Yangtze River	[10]
		993	1.35	12.8	90.0	Yangtze River	[3]
		19.2	N.D.	5.89	94.4	Sangong River	[7]
**Macrolides**	**MLs**						
Azithromycin	AZM	49.5	N.D.	11.8	67.1	North Canal	Present study
		41.6	3.12	20.2	100	Xiaoqing River	[4]
		6.64	N.D.	2.02	57.1	Yangtze River	[10]
		56.7	N.D.	2.07	94.0	Yellow River	[11]
		3.60	-	2.20	8.00	Lake Vanern, Lake Vattern, and Lake Malaren	[6]
Clarithromycin	CLR	83.2	N.D.	20.9	98.8	North Canal	Present study
		47.6	1.25	22.0	100	Xiaoqing River	[4]
		2.93	N.D.	0.46	28.6	Yangtze River	[10]
		N.D.	N.D.	N.D.	0.00	Sangong River	[7]
		0.73	-	0.53	8.00	Lake Vanern, Lake Vattern, and Lake Malaren	[6]
Roxithromycin	ROX	47.8	N.D.	12.7	97.6	North Canal	Present study
		176	7.37	79.4	100	Xiaoqing River	[4]
		4.77	N.D.	0.83	35.7	Yangtze River	[10]
		111	1.22	15.8	100	Dongting Lake	[12]
		< MDL	< MDL	< MDL	0.00	Lake Vanern, Lake Vattern, and Lake Malaren	[6]
Erythromycin	ERY	29.6	N.D.	4.42	86.6	North Canal	Present study
		46.6	1.42	17.1	100	Xiaoqing River	[4]
		200	N.D.	7.01	12.0	Chao Lake	[5]
		40.2	0.83	10.0	100	Liao River	[1]
		12.0	-	3.50	22.0	Lake Vanern, Lake Vattern, and Lake Malaren	[6]
**OTs**	**-**						
Lincomycin hydrochloride	LIN	45.8	N.D.	5.74	62.2	North Canal	Present study
		8.15	2.92	5.29	100	Yangtze River	[10]
		216	0.22	29.5	85.0	Yangtze River	[3]
		11.5	N.D.	4.48	94.4	Sangong River	[7]
Chloramphenicol	CP	32.3	N.D.	1.18	47.6	North Canal	Present study
		4.40	N.D.	0.90	60.0	Yangtze River	[3]
		15.4	4.1	4.6	100	Karst River	[13]
		218	14.9	80.9	68.6	Arkavathi Basin	[14]
Ampicillin	AMP	N.D.	N.D.	N.D.	0.00	North Canal	Present study
		892	N.D.	22.8	4.00	Chao Lake	[5]
		14.9	N.D.	4.86	81.3	Huai River	[15]
		10.2	N.D.	3.51	76	Yellow River	[15]
Florfenicol	FFC	139	N.D.	3.55	76.8	North Canal	Present study
		34.2	N.D.	3.30	95.0	Yangtze River	[3]
		3.60	N.D.	1.54	94.4	Sangong River	[7]
Gemfibrozil	GEM	14.8	N.D.	0.48	4.88	North Canal	Present study
		1.06	N.D.	0.16	76.0	Yellow River	[11]
		229	2.70	-	65.0	Paraopeba River	[16]
		< MDL	< MDL	< MDL	0.00	Lake Vanern, Lake Vattern, and Lake Malaren	[6]
Carbamazepine	CBZ	18.7	N.D.	6.02	91.5	North Canal	Present study
		7.92	0.74	3.37	100	Yellow River	[11]
		5.63	2.19	3.25	100	Huai River	[15]
		8.32	2.71	4.82	100	Yellow River	[15]

Pharmaceuticals	Abb	Max (ng/L)	Min (ng/L)	Mean (ng/L)	DF (%)	Region	Reference
Diclofenac	DF	37.0	-	6.60	100	Lake Vanern, Lake Vattern, and Lake Malaren	[6]
		54.8	N.D.	15.3	92.7	North Canal	Present study
		1130	202	545	28.6	Arkavathi Basin	[14]
		561	6.30	-	30	Paraopeba River	[16]
		552	N.D.	204	-	Zhangxi River	[17]
		359	N.D.	131	-	Lu River	[17]
Ritonavir	RTV	23.0	-	6.10	27.0	Lake Vanern, Lake Vattern, and Lake Malaren	[6]
		18.0	N.D.	1.64	59.8	North Canal	Present study
		91.7	N.D.	4.37	0.05	Llobregat River	[18]
		10.0	N.D.	1.53	41.0	Rivers in Wuhan	[19]

Table S6. PNEC values of pharmaceuticals and calculated method.

**Pharmaceuticals**	**PNEC (ng/L)**	**Method**
SPD	10000	AF
TMP	29	AF
SMM	1.20	AF
SMZ	11900	AF
SDA	2700	AF
SMX	8660	SSD
SMZ-2	30000	AF
OTC	7580	SSD
DOXY	2000	AF
TC	7550	SSD
OFX	40	AF
EFX	32000	AF
CIP	40	AF
NFX	160	AF
PEF	9220000	AF
AZM	480	AF
CLR	2	AF
ROX	66	AF
ERY	200	AF
LIN	3000	AF
CP	130	AF
AMP	0.31	AF
BF	0.68	AF
FFC	2300	AF
CA	2740	SSD
GEM	3060	SSD
CBZ	2070	AF
DF	749	SSD
RTV	2.90	ECOSAR

Table S7. List of abbreviations used in the manuscript except pharmaceuticals.

**Abbr.**	**Full title**
WWTP	Wastewater treatment plants
MDL	Method detection limits
PMF	Positive matrix factorization
EPA	U.S. Environmental Protection Agency
RQ	Risk quotient
MEC	Measured environmental concentration
PNEC	predicted no-effect concentration
SSD	Species sensitivity distribution
AF	Assessment factor
ECOSAR	Ecological structure activity relationships
EC50	The median effective concentration
NOEC	No observed effect concentration
COVID-19	Coronavirus disease 2019

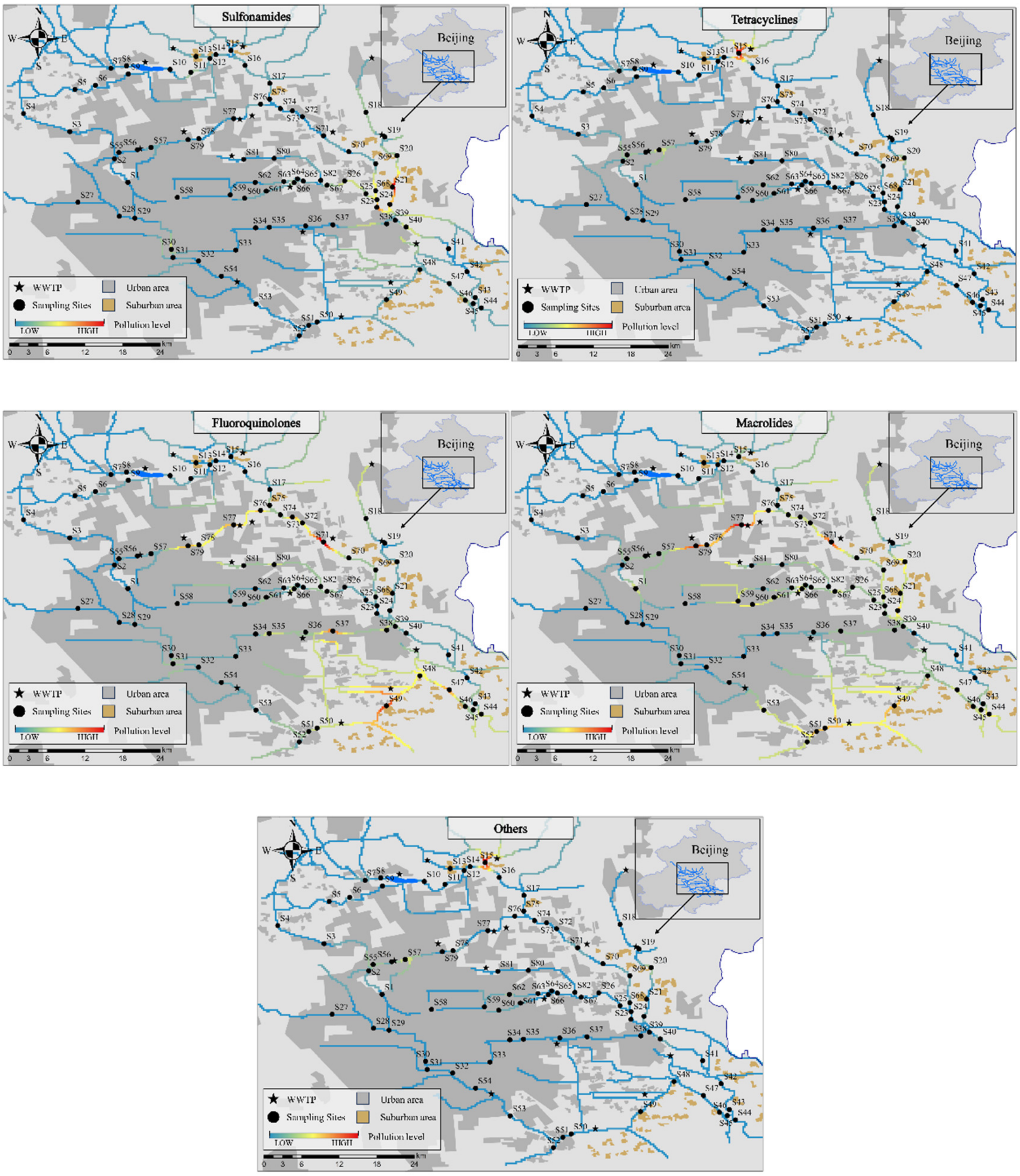

Figure S1. Interpolation map of inverse distance weights of each pharmaceutical concentration category in the North Canal Basin.

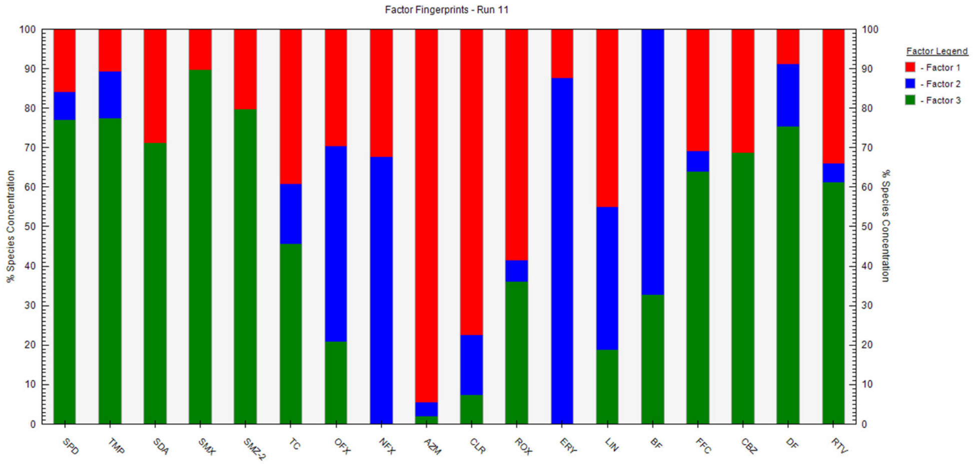

Figure S2. The contributions of all the identified sources to the pharmaceuticals in the North Canal Basin.

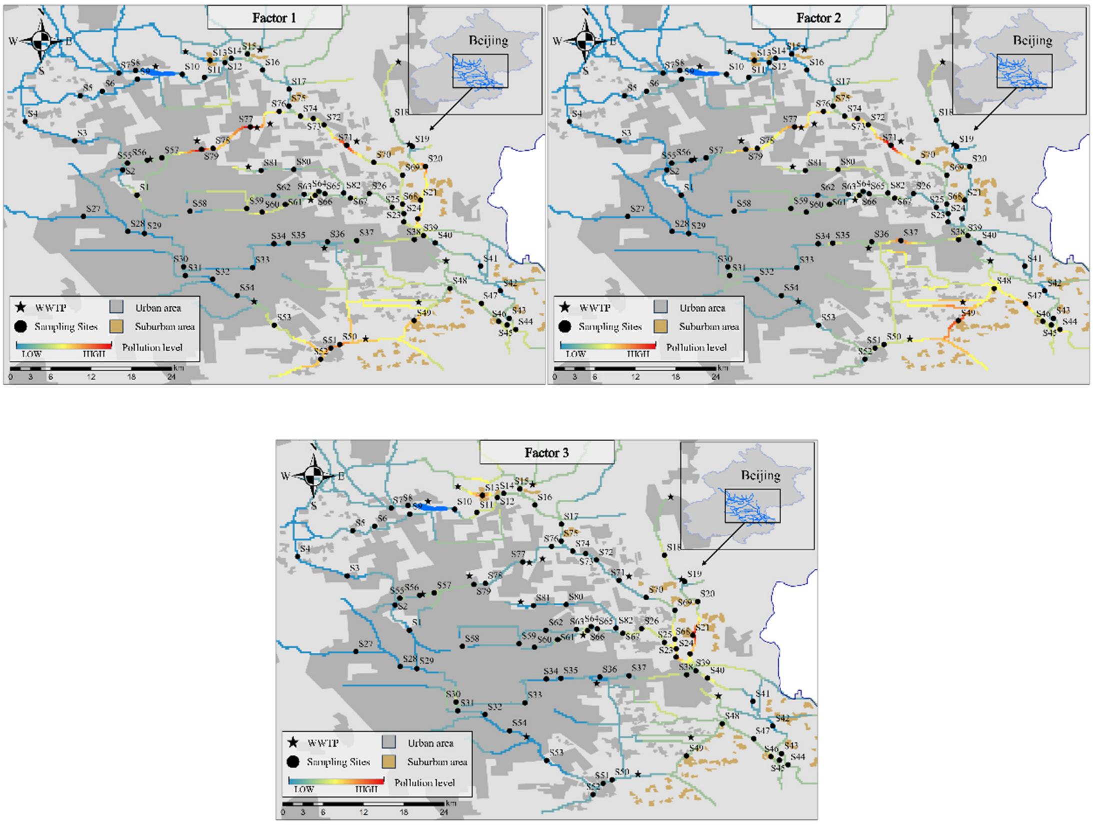

Figure S3. Interpolation map of inverse distance weights of each pharmaceutical concentration factor in the North Canal Basin.

Reference

1. Gao, H.; Zhao, F.; Li, R.; Jin, S.; Zhang, H.; Zhang, K.; Li, S.; Shu, Q.; Na, G. Occurrence and Distribution of Antibiotics and Antibiotic Resistance Genes in Water of Liaohe River Basin, China. *J. Environ. Chem. Eng.* **2022**, *10*, 108297, doi:10.1016/j.jece.2022.108297.
2. Linghu, K.; Wu, Q.; Zhang, J.; Wang, Z.; Zeng, J.; Gao, S. Occurrence, Distribution and Ecological Risk Assessment of Antibiotics in Nanming River: Contribution from Wastewater Treatment Plant and Implications of Urban River Syndrome. *Process Saf. Environ. Prot.* **2023**, *169*, 428–436, doi:10.1016/j.psep.2022.11.025.
3. Guo, F.; Wang, Y.; Peng, J.; Huang, H.; Tu, X.; Zhao, H.; Zhan, N.; Rao, Z.; Zhao, G.; Yang, H. Occurrence, Distribution, and Risk Assessment of Antibiotics in the Aquatic Environment of the Karst Plateau Wetland of Yangtze River Basin, Southwestern China. *Int. J. Environ. Res. Public. Health* **2022**, *19*, 7211, doi:10.3390/ijerph19127211.
4. Ci, M.; Zhang, G.; Yan, X.; Dong, W.; Xu, W.; Wang, W.; Fan, Y. Occurrence of Antibiotics in the Xiaoqing River Basin and Antibiotic Source Contribution-a Case Study of Jinan City, China. *Environ. Sci. Pollut. Res.* **2021**, *28*, 25241–25254, doi:10.1007/s11356-020-12202-z.
5. Zhou, Q.; Liu, G.; Arif, M.; Shi, X.; Wang, S. Occurrence and Risk Assessment of Antibiotics in the Surface Water of Chaohu Lake and Its Tributaries in China. *Sci. Total Environ.* **2022**, *807*, 151040, doi:10.1016/j.scitotenv.2021.151040.
6. Malnes, D.; Ahrens, L.; Köhler, S.; Forsberg, M.; Golovko, O. Occurrence and Mass Flows of Contaminants of Emerging Concern (CECs) in Sweden's Three Largest Lakes and Associated Rivers. *Chemosphere* **2022**, *294*, 133825, doi:10.1016/j.chemosphere.2022.133825.
7. Wu, S.; Hua, P.; Gui, D.; Zhang, J.; Ying, G.; Krebs, P. Occurrences, Transport Drivers, and Risk Assessments of Antibiotics in Typical Oasis Surface and Groundwater. *Water Res.* **2022**, *225*, 119138, doi:10.1016/j.watres.2022.119138.
8. Deng, W.-J.; Li, N.; Ying, G.-G. Antibiotic Distribution, Risk Assessment, and Microbial Diversity in River Water and Sediment in Hong Kong. *Environ. Geochem. Health* **2018**, *40*, 2191–2203, doi:10.1007/s10653-018-0092-1.
9. Ding, H.; Wu, Y.; Zhang, W.; Zhong, J.; Lou, Q.; Yang, P.; Fang, Y. Occurrence, Distribution, and Risk Assessment of Antibiotics in the Surface Water of Poyang Lake, the Largest Freshwater Lake in China. *Chemosphere* **2017**, *184*, 137–147, doi:10.1016/j.chemosphere.2017.05.148.
10. Liu, Y.; Chen, Y.; Feng, M.; Chen, J.; Shen, W.; Zhang, S. Occurrence of Antibiotics and Antibiotic Resistance Genes and Their Correlations in River-Type Drinking Water Source, China. *Environ. Sci. Pollut. Res.* **2021**, *28*, 42339–42352, doi:10.1007/s11356-021-13637-8.
11. Yu, X.; Yu, F.; Li, Z.; Zhan, J. Occurrence, Distribution, and Ecological Risk

Assessment of Pharmaceuticals and Personal Care Products in the Surface Water of the Middle and Lower Reaches of the Yellow River (Henan Section). *J. Hazard. Mater.* **2023**, *443*, 130369, doi:10.1016/j.jhazmat.2022.130369.
12. Guo, X.; Song, R.; Lu, S.; Liu, X.; Chen, J.; Wan, Z.; Bi, B. Multi-Media Occurrence of Antibiotics and Antibiotic Resistance Genes in East Dongting Lake. *Front. Environ. Sci.* **2022**, *10*, 866332, doi:10.3389/fenvs.2022.866332.
13. Zou, S.; Huang, F.; Chen, L.; Liu, F. The Occurrence and Distribution of Antibiotics in the Karst River System in Kaiyang, Southwest China. *Water Supply* **2018**, *18*, 2044–2052, doi:10.2166/ws.2018.026.
14. Gopal, C.M.; Bhat, K.; Ramaswamy, B.R.; Kumar, V.; Singhal, R.K.; Basu, H.; Udayashankar, H.N.; Vasantharaju, S.G.; Praveenkumarreddy, Y.; Shailesh; et al. Seasonal Occurrence and Risk Assessment of Pharmaceutical and Personal Care Products in Bengaluru Rivers and Lakes, India. *J. Environ. Chem. Eng.* **2021**, *9*, 105610, doi:10.1016/j.jece.2021.105610.
15. Feng, J.; Liu, Q.; Ru, X.; Xi, N.; Sun, J. Occurrence and Distribution of Priority Pharmaceuticals in the Yellow River and the Huai River in Henan, China. *Environ. Sci. Pollut. Res.* **2020**, *27*, 16816–16826, doi:10.1007/s11356-020-08131-6.
16. Corrêa, J.M.M.; Sanson, A.L.; Machado, C.F.; Aquino, S.F.; Afonso, R.J.C.F. Occurrence of Contaminants of Emerging Concern in Surface Waters from Paraopeba River Basin in Brazil: Seasonal Changes and Risk Assessment. *Environ. Sci. Pollut. Res.* **2021**, *28*, 30242–30254, doi:10.1007/s11356-021-12787-z.
17. Tang, J.; Sun, J.; Wang, W.; Yang, L.; Xu, Y. Pharmaceuticals in Two Watersheds in Eastern China and Their Ecological Risks. *Environ. Pollut.* **2021**, *277*, 116773, doi:10.1016/j.envpol.2021.116773.
18. Domínguez-García, P.; Rodríguez, R.R.; Barata, C.; Gómez-Canela, C. Presence and Toxicity of Drugs Used to Treat SARS-CoV-2 in Llobregat River, Catalonia, Spain. *Environ. Sci. Pollut. Res.* **2023**, *30*, 49487–49497, doi:10.1007/s11356-023-25512-9.
19. Zhang, Z. Impacts of COVID-19 Pandemic on the Aquatic Environment Associated with Disinfection Byproducts and Pharmaceuticals. *Sci. Total Environ.* **2022**.
